# Veterinary Expert: Legal Nature and Responsibility

**DOI:** 10.3390/ani13132163

**Published:** 2023-06-30

**Authors:** Andrzej Dzikowski

**Affiliations:** Department of Food Hygiene and Public Health Protection, Warsaw University of Life Sciences, 159 Nowoursynowska St., 02-787 Warsaw, Poland; andrzej_dzikowski@sggw.edu.pl

**Keywords:** veterinary ethics, veterinary forensic medicine, veterinary law, expertise, expert opinion, civil law, penal law, legal procedure

## Abstract

**Simple Summary:**

The issuing of expert opinions is rarely found as a topic of discussion in veterinary colleges as one of the forms of veterinary practice. At the same time, it has a significant social, legal, and economic impact. This article examines veterinary experts and expertise from an interdisciplinary ethical and comparative legal perspective, regarding different definitions and underlying basic assumptions concerning the meaning of veterinary expertise. Analysis reveals how the terminology used changes the perception of the examined institution. Legally relevant factors of an expert opinion are identified: direct connection with the law and the intended, applied use of this opinion in a legal context. A variety of sources of legal and ethical duties and the related liability of the veterinary expert are revealed, both as a proficient and as a member of a public trust profession. Different theoretical and practical suggestions are made to clarify and facilitate expertise in various contexts of veterinary professional practice.

**Abstract:**

Veterinary professional practice can be performed in many forms, including expert activity. The importance of veterinary expertise is, however, often underrated and limited to only one of its areas. Nonetheless, veterinary expert opinions have significant social, legal, and economic impacts. This study investigates veterinary expertise from an interdisciplinary, comparative perspective. Ethical and legal analysis and interpretation are performed. Essential concepts and relevant aspects of veterinary expertise are analysed. Legally relevant factors of an expert opinion are identified. The relationship between the law, the language, and the understanding of the role and duties of a veterinary proficient is demonstrated. A variety of possible expert opinions and the multiplicity of veterinary scopes of such activity is presented. It is argued that the ranges of forensic veterinary medicine and of veterinary expertise are broader than is predominantly assumed. Veterinary forensic medicine is a crucial part of veterinary specialisation. Ethical and legal basics, and the scope of veterinary expert’s liability, are revealed and discussed. The conclusion is that the duties and responsibilities of expert veterinarians are particularly great due to the exercise of the public trust profession, with large importance for the whole society. Their observance is, however, crucial to ensure the highest quality of expert opinions issued by veterinarians.

## 1. Introduction

Among the forms of veterinary professional practice, the issuing of decisions, predicates, certificates, reports, and opinions of various kinds can be indicated [1,2]. Not only decisions on the state of health of an animal, which are the everyday work of general practitioners, but also expert opinions belong to this sphere of professional activity.

The general definition of an expert is based on the special skills or knowledge of a certain person [3,4,5,6,7,8,9,10,11,12,13]. Similarly, the definition of “expertise” could refer simply to a specific skill or type of knowledge [12]. These are abstract and globally consistent definitions. They are independent of a given jurisdiction and its specific, detailed legal regulations, or local traditions. Moreover, they are uniform and unanimous throughout science. This common, universal basis allows for the current analysis.

Any veterinary professional is, and should be, in fact, professional par excellence, and thus an expert in veterinary medicine [14,15,16]. He or she could specialise, with expertise in a particular scope of study, knowledge, and skills. Every veterinary surgeon is—or could be—an expert.

Therefore, a fundamental question may be asked about whether an expert includes

- any veterinary surgeon;
- a specialist, in the sense that a veterinarian deals with a given, specific problem area and has knowledge and skills in this respect;
- a specialist, in the sense of an officially recognised title, such as veterinary surgeon; or
- the legal function of a proficient?

The goal of the author is to analyse, prove, and present universal and essential features developed by science, professional ethics, and law worldwide, regardless of the jurisdiction. Such a general and universal type of study is possible due to the fundamental homogeneity of the considered problem, the essential identity of legal solutions on a global scale, and the uniformity of veterinary sciences.

The inevitability of interdisciplinary knowledge used in all types of veterinary practice, including expertise in all its meanings, is indicated [14,17]. Therefore, the current analysis has an interdisciplinary and comparative veterinary, legal, linguistic, and ethical nature. 

A comparative legal analysis of the concept of the veterinary expert is carried out. Nevertheless, it is decided to reduce the number of citations of legal acts to a minimum, selecting only the most representative ones. An etymological and linguistic analysis of the legal vocabulary also sheds light on the definitions accepted and adopted, and the background of the terms used. Functional and teleological paths of legal interpretation are also used.

## 2. Expert and Expertise

The definitions of “expert” and “expertise” are comprehensive and have existed since, at least, the second half of the 19th century [12], although the institution of expert veterinarians is much older [18]. It should be noted that the definitions of the discussed concepts are sometimes subject to logical fallacy, being defined as *idem per idem* (i.e., a vicious circle) [19].

Additional characteristics of an expert are emphasised, however, in the literature. According to the established opinions of the scientific community, a veterinary expert is a person qualified in veterinary medicine in terms of not only knowledge and skills but also his or her education, experience, training, and thus—personal cognizance [10,20,21,22]. The roles of long-term and ongoing practice, the current work at the time that the expert opinion is issued, experience in researching a given issue, and experience in issuing opinions are especially appreciated [9,19,21,22].

It should be noted that this covers not only any specific field of veterinary science or practice, but also a general veterinary medical cognizance, which laymen do not possess [1,6,10,12,16]. The point of reference is the state of the general, common knowledge of a typical citizen with an average education and no particular interests.

### 2.1. When a Veterinary Expertise Is Not Possible

It should be noted that veterinary peer courts, as a rule, should not commission expert opinions in veterinary sciences. As persons who possess special knowledge, the judges of such courts should assess the case investigated by themselves. According to the author, this is a specific variant of the *iura novit curia* (the court knows the laws) rule.

The only exception should be considered for highly specialised and rare aspects of veterinary medicine. Such a statement is an effect of the extensive teleological interpretation of the legal provisions, analogous to the possibility of admitting evidence from an expert opinion on international law before a court of justice [11].

On the other hand, if there was a judge in a civil or criminal court, who is a veterinary surgeon, he/she cannot use his/her personal veterinary knowledge and should order an expert opinion [11]. The accumulation of different procedural roles by the judge would violate the adversarial, contradictory nature of the court proceedings and would undermine the constitutional guarantees for the parties.

This conclusion does not apply only to exceptional situations in which the legislator has provided for the possibility of veterinarians acting as experts and arbitrators [23,24,25], and private arbitration at the request of the parties, where the veterinarian acts simultaneously as an expert and as a *iudex privatus* (private judge).

### 2.2. Scope and Impact of Veterinary Expertise

The veterinarian’s expert opinion can apply to all matters related to animals, as well as nature protection, environmental and ecosystem protection, interspecies interactions, public health protection, food safety, fodder, drugs, etc. [7,8,20,26,27,28].

A specific, underestimated type of veterinary experts is those who can be described as “scientific experts”. They could work on behalf of public authorities, government, non-governmental organisations, or industry, providing scientific veterinary expertise in biosecurity, food safety and public health, animal production and productivity, agriculture, etc. [12,15,16,28,29,30]. This includes expert opinions issued within state veterinary authorities, both carried out purely in the laboratory and as a result of “field work” with the principle of “learning by doing”, e.g., in the eradication of transmissible diseases [12,16,28,29]. This type of expertise may have an impact on society via the implementation and application of the scientific work and veterinary knowledge [12,28].

Another impact that veterinary expertise could have is its incorporation into the law-making process, at the local, national, and international levels (e.g., food safety laws, infectious disease combat and prevention, animal welfare, statutes regulating the economic use of animals and their productivity). As with the introduction of interdisciplinary elements of other sciences, including technical and ethics, into veterinary expertise [2,17,30], it is also possible and desirable to introduce elements of veterinary medicine, through expert opinions, into other technical sciences, humanities, and the law.

A very important area of expertise is the assessment of veterinary malpractice in civil and disciplinary proceedings [11,31].

## 3. Terminology Used and the Understanding of the Position and Duties of an Expert

In addition to the mentioned considerations, it is necessary to examine how the terminology used affects the (in)correct understanding of the legal institution, position, and duties of the veterinary expert.

### 3.1. Who Is an Expert?

The word “expert” is derived from the Latin *expertus*, *ex-peritus*. It is a designation of a person who is skilful or who is experienced in something. The word *experior*, likewise, means to try, to test, to put to the test, to make trial of a person or thing, to find out, to prove [32,33,34].

As already indicated, any veterinarian could be an expert in a legal sense [4,15]. Every veterinarian, including general practitioners, is a specialist in his field and is recognised as such by the law. As has been stated above, all veterinary knowledge is a special factor that laymen do not possess [15,16].

Veterinarians could practice issuing “opinions”, even unknowingly or unaware [8], but this does not imply that every aspect of veterinary practice is expertise. The factors that make a given activity an expert opinion will be presented in the following part of the analysis.

The conducted research allowed the conclusion that the term “expert witness” is widely abused [35,36,37,38]. The nomenclature “expert witness” greatly (and faultily) influences the perceptions of experts in the Anglophone world. It should be observed that linguistic issues overlap in this respect with the legal regulations of the common law.

Many authors, as well as professional legislators, seem to be trapped in their linguistic imaginings, and—despite noticing and demonstrating fundamental differences—still place the expert opinion on par with evidence given by a factual witness [1,4,10,13,36,37,39].

There should be no doubt that the duties, powers, and the entirety of the legal institution of a veterinary expert differ significantly and fundamentally from cases in which a veterinary surgeon is called as a witness of a fact in a case. A veterinary expert’s opinion differs from factual evidence to the same extent. Indeed, these differences are so profound that they can be indicated without any doubt [10,13,36,37], so it was decided not to repeat these arguments in the current work.

#### 3.1.1. Professional Witness

Problematic, however, are the so-called “professional expert opinions”, as some [1,3], but not all [13], researchers refer to cases in which a veterinarian testifies before a court or other authority about information obtained in the course of his/her professional work. The equivalent term “professional witness’ evidence” is also used [1,3,10,35]. The activities of such a witness could include not only providing facts but also collecting evidence [9].

Notions are presented [1,9,10] according to which the “professional expert opinion” or “professional witness’ evidence” is a legal institution that lies between an expert opinion and a witness’ testimony. Supporters of this theory maintain that it is a specific form of a “*de facto* expert”, due to his/her professional qualifications.

This position is not only fundamentally errant but also internally contradictory. There should be no doubt that this is *nec plus ultra* the testimony of a factual witness (nothing further beyond). No particular knowledge is used directly in this testimony, and it is not the subject of evidence. The fact that the veterinary surgeon will use, in his testimony as a witness, the knowledge he/she has gained from education, practice, and experience is irrelevant. As with other witnesses, everyone has a certain degree of knowledge, background, and life experience. In the discussed case, the veterinarian is to provide only factual information. His/her profession has no particular legal relevance and is merely accidental.

#### 3.1.2. Professional Secrecy

Nevertheless, the witness testimony of a veterinary surgeon regarding any information obtained in the course of professional work is a very complex issue. It relates to one of the most important ethical and deontological obligations of a veterinarian, as a representative of a profession of public trust, namely professional secrecy [10,20,27,40,41].

This is one of the oldest duties of professional medical ethics, included in the Hippocratic oath [13,42]. It should be noted that professional secrecy applies to *all* information obtained during the work of a veterinarian. It applies to *all* facts, not merely the animal itself or its state of health or the animal’s owner. Moreover, it is not limited to clinical practice only and includes also official veterinarians and veterinary scientists.

A veterinarian may testify about the facts that he/she has gained as information in the course of his professional work only if he is released by an authorised body, such as the court, in the appropriate procedure. Alternatively, the person to whom the information relates may release the veterinary surgeon from confidentiality.

### 3.2. Further Terminology

A veterinary expert can also be named, particularly in the judicial context, a proficient. The word “proficient” is also derived from Latin, referring to somebody acting on behalf of somebody else [32,33,34].

It is the expert veterinary who conducts the examination, issues a decision, draws up a report, and presents it. He or she works for the layman, such as the judge. Expert opinion allows laymen to understand complex, specialised issues and problems. It is a particular form of assistance [1,4,6,10,19,22,26,31], and advice [1,4,6,14,15,32,43].

It has been found that in the veterinary scientific literature, the use of the aforementioned *terminus technicus* is extremely rare. The author hereby proposes the more frequent use of this term and the discontinuation of the usage of the ambiguous term “expert witness”.

Other terms used in a related sense include “connoisseur” (and “connoisseurship”), borrowed from French and used predominantly in relation to cuisine, music, and the fine arts, as well as the Italian term “cognoscente”, derived from Latin. Similar is the Yiddish-based “maven”. Etymologically, they all refer to someone who has specialised, above-average knowledge in a particular field [32,33,34]. A different, yet related, meaning is attributed to the word “doyen” (f. “doyenne”), meaning the oldest, and therefore the most experienced, individual [33]. Unlike the term “proficient”, their use in a veterinary context is not recommended.

As has been shown, most of the terminology is derived from Latin. A similar situation can be observed in most of the European languages. In other languages, particular terms referring to the expert carry the following semantic loads: knowledgeable, professionally competent, reasonable appraiser (e.g., German *Sachverständiger*), proper estimator, evaluator, adjudicator, adviser (e.g., German *Gutachter*), or somebody fluent and eloquent (e.g., Polish *biegły*).

### 3.3. Specialisation and Expertise

A “specialist” is a person devoted to a particular, specialised branch of science or a profession, particularly a medical one, such as a branch of veterinary medicine. The initial use of this term in English was in a medical context, similarly in French. Becoming specialised is a result of differentiation, adopting a specific area of study and practice, e.g., veterinary surgery, canine diseases, cariology, or neurology [44]. It should be noted that in most jurisdictions, there is no separate postgraduate specialisation in forensic veterinary medicine or forensic veterinary pathology [1,21,36,43].

Such specialised, above-average (compared to other veterinarians) knowledge and skills are the basis for being an expert—and, moreover, being one in an effective and correct way. They, however, do not in themselves make a given veterinarian legally proficient.

Synonymous terms “specialisation” and “expertise” are both used with different meanings, depending on the context [10,27], i.a., the specialised knowledge itself, the possession of such knowledge, the actual specialisation, the title of specialist, the administrative duties, and the performance of an expert opinion. The expert opinion itself can be also referred to as “expertise”.

Specialisation, or expertise, in a given field of veterinary medicine, e.g., in neurology, proves deepened professionalisation. It has a significantly positive impact on the cognizance of a given veterinarian [4,14] and increases their likelihood of examining a given type of case. The same must be said both for actual expertise in a certain field of veterinary medicine and for the officially recognised title of the specialised DVM.

On the other hand, it should be observed that specialisation in such a narrow sense is not a prerequisite and *sine qua non* condition for becoming a forensic veterinary expert [21]. As has been already mentioned, the reference point is the average level of a layman. The exact subject and complexity of the case are also important.

Doctoral and professor titles, as well as various training courses and scientific conferences, should be treated in a manner analogous to what was stated above for the legally recognised specialisation.

Indeed, the vast majority of veterinary experts are academics or specialised practitioners [1,21]. There are very few “experts by profession” who are devoted solely to examining cases and producing reports [1,21].

Nevertheless, a certain doctrinal consensus can be observed: actual skills and knowledge tested in practice are far more important than the title itself [7,9,19,45]. Such a certified title helps in obtaining a court order or a civil contract [1] and helps the expert to demonstrate his/her proficiency and professionalism [7]. Each diploma can be used as a credential, a certification of quality, and a justification of the competencies that one possesses in a tangible way [7,9,46].

In the case of a court expert, it is the court (the judge) who assesses the proficiency and decides and grants the status to the given veterinarian [7,11].

As has been revealed, above-average knowledge and skills are not, by themselves, enough. Being an expert in light of the law is also not tantamount to having the title of a specialist or actually specialising in a given branch of veterinary medicine.

### 3.4. Legally Relevant Factors

The above-mentioned question should be reposed, but from a slightly different point of view: what factors are legally relevant for the proficient or expert in the strictest sense?

The conducted research allowed the conclusion that these factors are

- a direct connection with the law, and
- the intended, applied use of the expert opinion or report in a legal context.

Therefore, for example, clinical examinations performed as part of the diagnostics and therapy of animals, post-slaughter examinations of meat, administrative examinations of district veterinary officers, or state veterinary hygienists and scientific tests do not provide an expert opinion *per se*. The listed factual and legal cases lack the simultaneous, cumulative occurrence of the two mentioned *sine qua non* prerequisites. In particular, administrative decisions regarding the suitability of meat for consumption, or designating an outbreak of an infectious animal disease, are not expert opinions. Such decisions are classified differently, due to being the fulfilment of administrative obligations imposed by the law.

### 3.5. Veterinary Expert and Veterinary Forensic Medicine

The term “veterinary forensic medicine” should also be understood similarly to the definition of the veterinary proficient. It covers any usage of veterinary medicine for the purpose of the law [1,36].

The author, however, strongly rejects the limitation of the legal institution of an expert to judiciary proceedings or to forensic pathology. Such a limitation would be contradicted by the arguments of both historical and current legal provisions on a global, comparative scale. One of the reasons for such a false, narrow understanding of the analysed issues is, as has already been shown, the abuse of the term “expert witness”.

Different types of expert opinions [4,11,17,36,39], together with the differentiation criteria, are presented in Table 1.

Henceforth, the legal context of veterinary medicine shall be broadly understood [5,36]: as court proceedings, administrative proceedings, legislation, contract conclusion, etc. Neither veterinary expertise nor veterinary forensic medicine are limited solely to court evidence or to pathology, as some hold [6,7,10,19].

Most of the veterinary literature focuses on forensic veterinary court experts in criminal proceedings [6,7,8,17,22,31,36,38,47,48,49], while other contexts of the activity of expert veterinarians are merely mentioned [1,2,5,7,26,31,44,48,50]. Animal abuse, animal welfare, animals as victims of accidents, animals causing injuries to people, and other issues related to pathomorphology provide relevant examples of veterinary expertise, but they are definitely not exhaustive.

Forensic veterinary medicine is a separate, broad, and interdisciplinary branch of science, and it is not the same as forensic veterinary pathology, which is its subdivision; a veterinary expert is not the same as a veterinary pathologist [8,14,17,36,47]. Nevertheless, according to the author, veterinary forensic medicine cannot be interpreted as synonymous with veterinary specialisation [5], the latter being a broader term.

In particular, attention should be paid to the private-law aspects of forensic veterinary medicine, and of veterinary expertise, such as animal-sales-related examinations and opinions. The author’s statement is based on the legal provisions of many legal regimes [31,51] and on the practical and economical significance of such expert opinions. This crucial theme is notoriously ignored in the majority of the veterinary scientific literature discussing expert opinions.

Moreover, the author’s view is supported by the linguistic interpretation, as the word “forensic” is derived from the Roman *forum* (market). Therefore, it is incorrect to claim that the extension of the meaning of forensic veterinary medicine (from forensic pathology to veterinary jurisprudence) is a trend of the recent past [1,26]. On the contrary, it is a return to the origins of the discussed discipline.

### 3.6. Untrue Novelty

It is often stated in the literature that veterinary forensic medicine is a new scientific discipline and that the activities of expert veterinarians are a “new form” of practicing the profession [1,8,47]. These are definitely erroneous and untrue statements of novelty.

The use of expert opinion in relation to veterinary medicine in its modern meaning in various normative acts dates back (at least) to the end of the 18th century. Eminent examples include expert veterinarians adjudicating on the occurrence of physical defects in animals, performing official examinations, and deciding on price reductions. Such regulations are, or have been, present in the following states: Austria, Belgium, Germany, France, Luxembourg, Poland, Spain, and Switzerland [23,24,25,46,52,53,54,55,56,57].

#### Sales-Related Expert Opinions

Bearing in mind the above examples, the author would like to return to the aforementioned sales-related veterinary examinations and issuing of sales-related expert opinions. It should be noted that the use of terms such as “vetting” or “pre-purchase examinations” [10,26,58,59,60,61] does not cover the entire scope of the issue in question.

These synonyms apply only to veterinary examinations conducted, and opinions issued, *before* concluding of the contract of the sale of an animal. Meanwhile, veterinary examinations are often carried out, and opinions issued, *after* the conclusion of the contract, *after* the animal’s delivery, when a physical defect in the animal is revealed and manifested. Examinations and opinions can be carried out both intravitally [17,26,36] and post-mortem [47], including post-slaughter.

There is no substantial difference between a veterinary examination aimed at issuing expert opinions before and after the conclusion of the contract, before and after the delivery, or those ordered by the seller, by the buyer, or by the court (in the event of a dispute regarding the animal warranty). German veterinary science provides a variety of names for sales-related veterinary examinations and opinions [57,58,59,60,61,62]; nevertheless, according to the author, they should not be differentiated [51,60].

A similar legal nature is presented in opinions issued by veterinarians after examining an animal’s health in relation to exhibiting it at competitions, shows, or races or allowing it to breed.

There is no doubt that the development of veterinary expertise and specialisation in the legal veterinary field has already taken place: in terms not only of the proper performance of the role of veterinary expert, and thus acting *lege artis*, but also legal obligations and professional ethics and deontology [51,57,58,59,60,61].

## 4. Characteristics of the Veterinary Expert Opinion

Unlike factual witnesses, who provide facts and information to the court, other authorities, or private persons, the expert witness provides an opinion: conclusions and interpretations of the results obtained [8,13].

It is reasonable to distinguish between a veterinary surgeon’s decision—a comprehensive verdict on an animal’s health status—and an expert opinion, also called a report. The first is a manifestation of the activity conducted by a clinician or pathologist, or a public veterinary officer. As has been revealed, decisions may be issued not only in the case of expert opinions.

The report/opinion is the final formulation of the results, observations, and conclusions of a proficient [51]. It should be noted that not all opinions of expert veterinarians are based on decisions on an animal’s health. Both decisions and reports may be presented in oral, written, or mixed form.

An opinion/report should be balanced, clear, complete, concise, confidential, considered (taking into account all data and materials, even those that detract from the conclusion), consistent with life experience, convenient, fact-based, impartial, independent, intelligible and understandable (to the layman), integral, in relation to the matter, justified, lawful, logical, methodologically appropriate, objective, precise, prepared within a certain period of time, professional, properly appointed and in accordance with the provisions of the procedure in question, qualified, relevant, reliable, scientifically based, scrupulous, self-confident, sound, truthful, unbiased, and uninfluenced [1,3,7,8,9,10,11,13,19,36,43]. It is especially important that the opinion is not written in an over-intellectualised or incomprehensible manner regarding its addressee, the layman [7,8,11,12,36]. The court, the public administration, the industry, and the animal owner must understand the opinion in order to benefit from it.

Opinion-making and the preceding examinations are dependent on the personal performance of the commissioned expert [11,51]. Acting by proxy or deputy is strictly prohibited, due to the strict personal nature of this obligation. As has been shown, the personal skills, knowledge, experience, and cognizance of a given proficient are the factors of key importance.

Moreover, the opinion/report should contain appropriate reservations, regarding, i.a., aspects that go beyond the scope of questions asked to the expert; that go beyond the scope of the specialisation, knowledge, skills, and competence of the expert; that go beyond the area, scope, or range of expertise; for which a decision cannot be made on the basis of the available research material or other insufficient data; and on which discrepancies or differing opinions are presented in the scientific discourse [8,9,10,13,21,40,50].

### Expert’s Liability

Therefore, the following opinions should be considered defective: those that are incomplete, those that go beyond the subjective or the objective, or the expert’s cognizance; and those that are illogical, internally contradictory, contradictory (in the case of several opinions), unscientific (contrary to current knowledge), illogical, intentionally falsified, unclear, methodologically incorrect, suggested, or biased [11].

In the case of court expert opinions, after analysing them in accordance with the principle of discretion (free assessment of evidence), the court accepts the opinion when it meets the requirements of correctness, rejects the opinion in its entirety, or partly rejects the opinion [4,19,22,48]. The court may also order a supplementary opinion (*ex officio*, or at the request of the party). The expert bears public-law liability for a faulty opinion, e.g., imprisonment or a pecuniary fine [11]. Penal liability for inappropriate behaviour in the courtroom is also possible [11]. The court expert is a public-law institution, and thus bears public-law liability of a criminal and administrative nature.

The issue of private, extra-judicial opinions is different. In the case of a defect in the opinion, the commissioning party (e.g., owner, seller, buyer of the animal) may not accept the defective opinion as a defective performance of the obligation (and, thus, a breach of contract), or they may possibly accept it. The commissioner is entitled to compensation, and, in some jurisdictions, also to a warranty for defects in the opinion. It is a civil, private-law liability [23,51,52,53,54,59,61,62], as the expertise commissioned in a civil contract has a private-law nature.

In addition, a veterinarian may be held professionally and disciplinarily liable in the case of defective opinions, both judicial and extra-judicial [51]. A veterinarian working in a scientific or state institution, or in a clinic, may also be liable as an employee [51].

Such a situation has, moreover, a significant negative effect on his/her scientific and professional reputation. His/her moral responsibility towards the whole society is, therefore, worth considering.

## 5. Duties of Veterinary Experts

The abstract, general, and unspecific obligations and duties of experts are well established both in legal acts—especially the codes of criminal, civil, and administrative procedures—and in judicial decisions, and the scientific literature. At the same time, these rules are the features of a proper expert opinion. Therefore, the same can be said about the duties of an expert [1]. These are interdependent and inextricably intertwined issues. Compliance with the expert’s duties results in the issuance of a proper opinion. All of these aspects are established and are uniform for all types of expert opinions; hence, they will not be discussed in more detail.

Instead, the author’s intention is to focus on a more complex problem: are the duties, responsibilities, and liability of a veterinary expert the same as the duties, responsibilities, and liability of proficients in other areas of expertise?

The conducted analysis allowed the conclusion that there are significant differences in the case of experts performing professions of public trust, such as veterinary medicine, and burdened with professional ethics [2,5,6,14,26,44,51,59,63,64]—in relation to experts who do not perform such professions and are not required to comply with professional ethics.

The duties of a veterinary professional and the duties of an expert overlap and complement each other. They arise from different sources and different systems of social control and result in different legal liabilities. In addition, there may be further mixing of duties derived from other sources, e.g., religion, national law, international law, labour law, civil law, scientific ethics, etc. [51]. All these conditions create a specific, individual, and uniform model of the bonds resting on a given entity [51].

Nevertheless, the premise that distinguishes the veterinary expert from other proficients is the professional ethics and deontology [13,26,51,64].

The risk of liability of the veterinary expert is, in the author’s opinion, greater than in other cases. Being a representative of the profession of public trust [57,62] is an additional criterion affecting the increase in the rigor of the liability and the lowering of its threshold.

Regardless of criminal and professional liability, a veterinary expert may also bear civil responsibility. In particular, the following civilian liability can be indicated: liability for damages, for *culpa in contrahendo* (fault in conclusion of the contract, in the case of private opinions), and tort liability [57,60,62], as well as liability towards third parties, e.g., an animal buyer who does not order an expert opinion [57,58,59,60,62].

It should be noted that in some jurisdictions, it is necessary for the court to notify the veterinary professional organisation and obtain its input (but not binding consent) regarding the person to be appointed as a proficient [45].

### 5.1. Secrecy and Documentation

One of the key ethical aspects is the obligation to maintain veterinary professional secrecy and confidentiality, as mentioned in the earlier part of the current study.

In addition, an obligation for any expert to maintain confidentiality related to *any* knowledge gained in the course of examination and the issuing of an opinion should be raised. The problem of the expert’s confidentiality is crucial in terms of the rule of law and is often present in normative acts, but it is considered extremely rarely in the veterinary literature [10,11,45]. According to the author, this gap in the veterinary literature may be caused by the “natural” conviction of veterinary surgeons about the need to maintain secrecy.

While the two legal obligations of secrecy overlap in the discussed area of veterinary expert opinions, in other areas of expertise, this regularity is not observed due to the lack of the ethical principle of professional secrecy. Meanwhile, the obligation of veterinary ethics is broader and more general than that of any expert. The former includes, as has already been demonstrated, *any* knowledge gained in the course of *any* professional activity of *any* veterinary surgeon, including information gained as a proficient. One should, however, bear in mind that the legal basis of these two overlapping obligations is different.

Another very important duty, associated with secrecy, to be guided by the veterinary proficient, is documentation. It concerns the entire course of the expert’s study, the results of all analyses and tests conducted, as well as the report, as the final summary of the expertise. This obligation has its sources in professional law (veterinary medical documentation, possibly also concerning informed consent), in court procedural law, and in civil law (proper performance of the obligation, due diligence) [4,10,20,27,40,41,51].

### 5.2. Independency and Advocacy

It is argued that the expert veterinarian should remain independent of any influence and never assume the role of an advocate [8,9,13]. This undoubtedly applies to the parties to the court proceedings, such as the accused, defendant, or claimant [10]. Regardless of whether the expert was appointed at the request of one of the parties to the proceedings, or *ex officio* or *motu proprio*, he/she appears before the court and for the court. Furthermore, the principle of independency applies to certain influence groups, politicians, social organisations, relatives, friends, representatives of certain views, etc. The rule of objectivity includes also the deontological prohibition of issuing opinions in relation to one’s own clinical cases, owned animals, or enterprises [20,27].

A provocative question could be, however, posed: does this also apply to animal advocacy?

In order to answer this question correctly, one should set aside specific legal regulations and one’s own views and prejudices and try to look at the problem in a general and holistic way. The research carried out made it possible to present a functional interpretation of the discussed problem.

It is a well-known fact that animal advocacy is the highest ethical and deontological rule of the veterinary profession in many jurisdictions, especially Anglo-Saxon ones [10,13,14,40,41], but, nowise, in all. According to some regulations [20], the most important rule in the veterinary profession is to ensure the welfare of mankind, while other professional veterinary organisations do not indicate any primary rule *explicite*.

Consequently, does a veterinarian presenting an expert opinion in which, in order to maintain impartiality, independency, and objectivity, he/she evades assessment based on animal advocacy or another guiding rule and acts against the law, deontology, and ethics of a professional corporation? The answer is no. As has been shown, the primary duty of the expert is to maintain objectivity and impartiality, and the primary role of the expert is to provide independent, substantive assistance. Therefore, an expert cannot adopt any point of view that favours any side of the proceedings, on the basis of animal advocacy or the good of mankind.

Nevertheless, he/she may and should include in his/her opinion a statement that, in accordance with the prevailing rule of professional ethics, the case analysed in the opinion takes a certain rating. The aforementioned rule is also the part of specific knowledge of the expert. It is the court (or the other authority ordering the opinion) that proceeds to its comprehensive and free assessment.

The case of private veterinary opinions is slightly different; hence, the expert veterinarian is afforded greater freedom of expression but should always indicate the ethical basis for his/her claim.

Thus, another principle of veterinary professional ethics is also implemented: the exclusion and disclosure of any possible conflicts of interest [3,10,14,20,40]. As has been already mentioned, this deontological imperative applies primarily to one’s own clinical cases and owned animals [27]. According to the author, such a legal and ethical provision implements the general principle of law: *nemo iudex in causa sua* (no-one is judge in his own cause).

### 5.3. Cognizance and Correctness

Another important aspect of the expert’s duties, and of the characteristics of the opinion, which the author would like to raise, is basing the opinion on scientific grounds, methodological correctness, and the use of appropriate research methods [13]. This is the implementation of another important requirement and professional rule applicable to all veterinarians: the use of current medical knowledge [10,20,27,40,41]. This statement is related to another deontological obligation of each veterinarian, continuous self-education as a part of the *lege artis* practice [11,14,20,65]. The provision of specialised knowledge in a given field does not, however, imply the veterinary medical training of the person, or authority, that commissioned the expert’s opinion [66].

A veterinary expert exceeding his/her own competences, expertise, the level of his/her own knowledge, skills, scientific competences, education, or professional experience, and the scope of the procedural competences expressed in the questions asked, will bear professional liability in the veterinary peer court [10,20,27,40,41,51].

Similarly, professional liability will be implied for malpractice and medical errors committed during the preparation of the opinion and the examination. Professional liability for malpractice is independent of the civil one (damages) [23,25,51,52,53,54,61,62].

Another eminent example of a deficiency implying professional liability is the commission of a crime by the expert, e.g., issuing a false opinion or bribery.

It should be noted that in some jurisdictions, appointing a veterinary surgeon as an expert by the court requires the veterinary professional corporation to be informed. The corporation should express its opinion (but not consent) in this matter [45].

### 5.4. Non-Universal Duties

Another issue worthy of deeper analysis is the collection of evidence by the veterinary expert. This part of the expert’s work is often pointed out in the literature on veterinary experts written in Anglophone circles [7,8,9,21,22,26,36,38,48], and thus in the common law context.

The obligation to collect evidence, inspect the crime scene, conduct the chain of custody, and other resulting responsibilities [7,9,10,21,22,26,36,38,48,49,67,68], and liability for non-compliance with the law, results from the Anglo-Saxon penal law system, including the offices of a coroner or medical examiner.

In continental law, the situation is completely different. Statutory provisions explicitly or implicitly prohibit the collection of evidence and sampling by any experts, including veterinarians [11]. Under such provisions, the expert may examine only evidence (e.g., animals, animal corpses, case files, documents, laboratory test results) provided by procedural entities, such as the police, or the prosecutor.

A significant exception to this prohibition has been developed in the legal doctrine and jurisprudence. Thus, collecting additional evidence in the examined material is possible in the case of the threat of destruction of this evidence, or the impossibility of its subsequent analysis [11]. It is the result of the systemic and teleological interpretation of the law, with which the author does agree and affirm.

The discussed essential legal difference between continental and common law countries is not reported in the global veterinary literature. It has been discussed only in local analyses, focused on the legal system of the particular state, usually in non-congress languages [11,64]. In the author’s opinion, therefore, this problem needs to be analysed and discussed in the current study. Hereby, significant differences in the court procedure, duties, and empowerment of the expert veterinarian in different legal systems are revealed, as well as fundamental disparities in the judicial proceedings. The issue of the contradictory nature of the trial in the common law system, and the behaviour in the courtroom related to it, should be assessed in an analogous way.

The discussed differences significantly affect the course of expertise, as well as the duties of veterinary experts.

### 5.5. Veterinary Experts’ Legal Knowledge

It should be observed that legal knowledge is a legal requirement for an expert in all types of legal systems [26,31,45]. Moreover, it is also a legal and deontological obligation of every veterinarian [10,17,20,26,27,41]. It is therefore, as analysed above, a duty for veterinary experts of a double nature, double source, and broad, multifarious liability. The importance of legal awareness in this regard among veterinary surgeons is revealed, especially in the face of progressive globalisation and cases of appointing outstanding, foreign veterinarians as experts.

### 5.6. Wide Range of Responsibility

The responsibility of the veterinary expert, as a professional *par excellence* and a member of a public trust profession, to the general public can be derived from ethical codes [6,10,14,20,27,41]. The moral responsibility of all experts and of all scientists can also be assumed, but there is no way to respect it directly, unlike the morality of veterinarians, the violation of which is punishable by professional liability before peer tribunals.

Modern changes in humans and humans in relation to animals bring about changes in public morality. These are accompanied by changes in the law. Changes in the professional veterinary ethics, including the strengthening of the obligations of expert veterinarians to maintain a level of due diligence in their practice, are implied. Social implications, public expectations, and inter-systemic connections require new tasks, behaviours, and approaches from veterinarians [14,43]. The development of the professional activities of veterinarians within their expertise is associated with a rise in the level of responsibility, and liability—primarily ethical and civil.

## 6. Conclusions

Proficiency and expertise—the comprehensive work of an expert veterinarian—are crucial from theoretical, practical, legal, and veterinary points of view. It is a significant form of veterinary practice. It should be emphasised that veterinary forensic medicine has been found to be an important part of the complex phenomenon of veterinary specialisation.

It has been found that the role of a veterinary expert can be defined as the legal function of a proficient: a veterinary surgeon whose substantive statement (opinion/report) is based on specific veterinary knowledge and skills and meets the legally relevant criteria: a direct connection with the law and the intended, applied use of this opinion in a legal context.

It has been revealed that the diversity of terminological forms significantly affects the correct and incorrect understanding of the institution, role, tasks, and responsibilities of veterinary experts. It has been shown that limiting oneself to only one legal system, without comparative studies, significantly narrows the understanding of the discussed problem. Moreover, it has been proven that the global view allows for a uniform and consistent foundation for all veterinarians who will act as expert witnesses in the future.

It has been revealed that the problem of the responsibility of a veterinary expert for the, broadly understood, defectiveness of judgments and conclusions issued by him/her on the basis of the conducted examination is complex and multifaceted. The basis of this complexity is, above all, the multitude and complexity of the obligations imposed on the veterinarian providing the expertise. The origin of these obligations (from different, but complementary, source systems of values and obligations) is not without significance.

The development of the practice and theory of the analysed problem has had a significant impact on the development of veterinary medicine, especially in terms of research procedures and of specifying the standards of due diligence and the compliance of conduct with ethical and deontological principles.

Trends for the future in veterinary expertise are expected to gain the interest of both public and private commissioners, which corresponds to an increase in the frequency of disputes [1,43,47]. This applies to evaluating the malpractice and unethical behaviour of other veterinarians as well [43].

An increase in the importance of veterinary expertise is expected, in terms of not only legal matters but also in the further development of veterinary medicine—as a science, and as an ethical system of professional duties [14]. Thus far, the predictions have been realised [1,43]; it is, therefore, expected that these processes will continue in the future.

In particular, it may be important to intensify the use veterinary proficient opinions in legislative processes and in economic development, through the use of interdisciplinary knowledge and skills and the navigation of public discourse in animal matters.

The analysis conducted indicates that the ultimate goal of litigation, as well as of scientific research and of issuing expert opinions, is to discover the truth. This brings the court trial and the expert opinion formation process closer to the research and the publication of its results in science. Axiology and praxeology, as well as professional and social liability, are analogous and comparable in these areas of veterinary professional activity.

## Figures and Tables

**Table 1:** Types of expert opinions.

Criteria	Types of Opinions	
Commissioning entity	Court	Prepared by registered experts
		Prepared by experts appointed ad hoc or *ad casum*
		Commissioned *ex officio*/*motu proprio*
		Commissioned at the request of the party to the proceedings
	Official (ordered by the police, procurature, public authority)	
	Private, extrajudicial (such opinion may be used in the court as a substantive support, source of arguments, source of further evidence requests, motion or request for an evidence, private document evidence, basis for a supplementary opinion of another expert, or for appointing an expert, etc.)	
Mandatority/not	Mandatory (required by the law)	
	Facultative	
Form of realisation	Independent, individual expert	
	Collective (prepared by two or more veterinary surgeons) and complex (one opinion prepared by the veterinary expert and expert(s) from another field of expertise)	
	Institutional (prepared by scientific institutions, public veterinary administrative institutions, veterinary clinics, or veterinary laboratories; form excluded in some jurisdictions, yet very useful in veterinary science, where elaborate opinions that require equipment and many specialists can be developed)	
Content of the opinion/report	*Sensu largo* (containing course of activities, examinations, and conclusions, as well as additional documents, e.g., results of laboratory tests)	
	*Sensu stricto* (solely conclusions, allowed predominantly in the course of purely clinical or administrative practice)	
Form of the opinion/report	Oral	
	Written	
	Mixed	
Type of material	Abstract (theoretical)	
	Examination-based	
	Based on case files	
	Based on case files and examination	
Order, task assigned, or importance	Primordial	
	Ultimate	
	Partial	Preliminary
		Provisional
		Supplementary (by the same expert as the primordial one)
		Accessory
	Counter-expertise (by another veterinary expert besides the primordial one, having the same value as evidence)	
	Super-expertise (in German: *Oberexpertise*; improperly named “consulting expertise” by some authors; by other veterinary experts besides the previous expert(s), evaluating previous opinion(s), having higher value as evidence; type present only in some jurisdictions, such as Switzerland, while in others it is explicitly excluded by the law)	
Type of expert’s conclusion or justification	Categorical (unequivocal)	
	Probable (alternate)	
Content of expert’s conclusion or justification	Total	
	Partial	

## Data Availability

Not applicable.
